# Sex-Related Online Behaviors, Perceived Peer Norms and Adolescents’ Experience with Sexual Behavior: Testing an Integrative Model

**DOI:** 10.1371/journal.pone.0127787

**Published:** 2015-06-18

**Authors:** Suzan M. Doornwaard, Tom F. M. ter Bogt, Ellen Reitz, Regina J. J. M. van den Eijnden

**Affiliations:** 1 Department of Interdisciplinary Social Science, Utrecht University, Utrecht, the Netherlands; 2 Utrecht Centre for Child and Adolescent Studies, Utrecht University, Utrecht, the Netherlands; Leibniz Institute for Prevention Research and Epidemiology (BIPS), GERMANY

## Abstract

Research on the role of sex-related Internet use in adolescents’ sexual development has often isolated the Internet and online behaviors from other, offline influencing factors in adolescents’ lives, such as processes in the peer domain. The aim of this study was to test an integrative model explaining how receptive (i.e., use of sexually explicit Internet material [SEIM]) and interactive (i.e., use of social networking sites [SNS]) sex-related online behaviors interrelate with perceived peer norms in predicting adolescents’ experience with sexual behavior. Structural equation modeling on longitudinal data from 1,132 Dutch adolescents (M_age_ T_1_ = 13.95; range 11-17; 52.7% boys) demonstrated concurrent, direct, and indirect effects between sex-related online behaviors, perceived peer norms, and experience with sexual behavior. SEIM use (among boys) and SNS use (among boys and girls) predicted increases in adolescents’ perceptions of peer approval of sexual behavior and/or in their estimates of the numbers of sexually active peers. These perceptions, in turn, predicted increases in adolescents’ level of experience with sexual behavior at the end of the study. Boys’ SNS use also directly predicted increased levels of experience with sexual behavior. These findings highlight the need for multisystemic research and intervention development to promote adolescents’ sexual health.

## Introduction

Over the past decade, a growing body of research from various parts of the world has addressed the role of sex-related online behaviors in adolescents’ sexual development. Sex-related online behaviors refer to the use of the Internet for activities revolving around sexually tinted arousal/entertainment, information-seeking, communication, exploration, self-portrayal, and cybersex [1, 2]. Such behaviors can be receptive, communicating sexual content one-way from medium to user, or interactive, enabling users to create, distribute, and comment on sexual content. In the receptive category, adolescents’ use of sexually explicit internet material (SEIM) has received particular attention, and a substantial number of studies have sought to document the attitudinal, emotional, and behavioral consequences of exposure to this material (for a review, see [3]). With regard to interactive online behaviors, Social Networking Sites (SNS) have recently been researched as potentially powerful platforms for adolescents to form and evaluate conceptions of sexuality and sexual attractiveness, as well as to experiment with and portray one’s sexual identity [4–6]. Unlike SEIM use, SNS use is a social activity that is not explicitly sexual in genre; most adolescents do not engage in this behavior for the purpose of seeking exposure to sexual content. Nonetheless, as several studies [e.g., 4–6] have pointed out, when using SNSs adolescents may be exposed to sex-related messages by peers, engage in sexual communication with other users, or create and distribute sex-related content themselves. Evidence to date indicates that SEIM use and SNS use predict various aspects of adolescents’ developing sexuality. These include more permissive and instrumental attitudes toward sex [7–9], less satisfaction with one’s sexual experience [2, 10], more body surveillance and body image concerns [2, 11, 12], and earlier and more advanced experience with sexual behavior [7, 8].

However, apart from what they predict, much less is known about how these sex-related online behaviors shape adolescents’ sexual development. Remarkably, studies on the effects of sex-related Internet use have often isolated the Internet and online behavior from other, offline processes in young people’s lives [13, 14]. This is in contrast with prominent ecological and multisystemic approaches–such as Bronfenbrenner’s [15] Ecological Systems Theory–that conceptualize sexual development as the outcome of multiple influencing and interrelating systems [16]. Among the multiple systems of influence in adolescents’ lives, peers are considered to be of particular importance. During adolescence, young people spend large amounts of time with their friends, and they put substantial value on the expectations and opinions of peers [17, 18]. Consistent with this notion, meta-analytic evidence has indicated that perceived peer norms regarding sexuality strongly guide adolescents’ sexual decision-making. Specifically, perceptions of peers’ approval of sexual behavior (i.e., injunctive norms) and perceptions of peers’ sexual behavior (i.e., descriptive norms) have been found to predict adolescents’ own sexual activity [19].

Given the increasing engagement with both the Internet and peers during adolescence [17, 18, 20] and the fact that some online behaviors–particularly interactive behaviors as SNS use–take place at least partly in a peer context, it seems necessary that research takes an integrative approach to better understand how these systems interrelate and combine in shaping adolescents’ sexual development. Drawing on key theories in the domains of media and peer effects, the goal of the current study was to test an integrative model explaining how two sex-related online behaviors (i.e., SEIM use and SNS use) are linked to perceived peer norms in predicting adolescents’ experience with real-life sexual behavior.

### Integrative model of sex-related online behaviors and perceived peer norms

**Fig 1:**
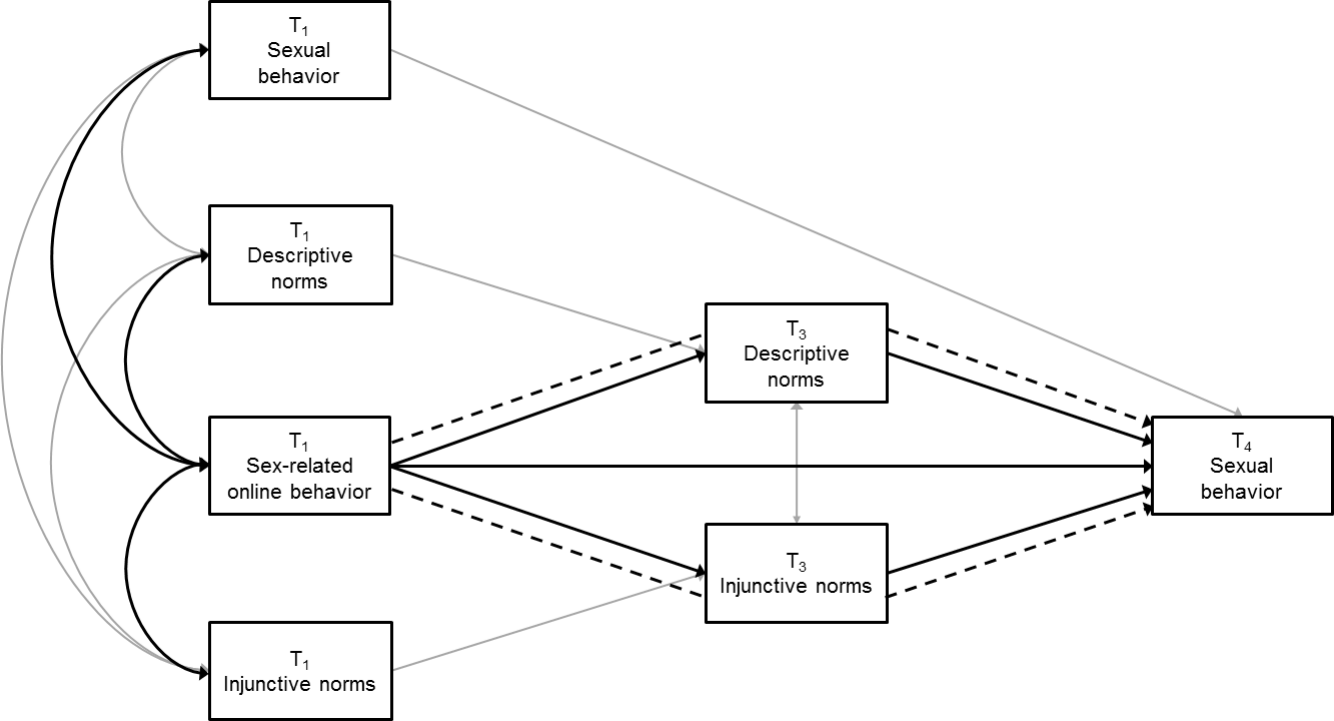
Integrative model of sex-related online behaviors, perceived peer norms, and sexual behavior. Bold construct and arrows represent the tested hypotheses.

Fig 1 shows an integrative model of how receptive and interactive sex-related online behaviors and perceived peer norms may interrelate to predict adolescents’ experience with sexual behavior. The arrows represent the various theoretical assumptions on which the model is built. As becomes clear, the model hypothesizes three types of relations among sex-related online behaviors, perceived peer norms, and sexual behavior: (a) baseline associations, (b) direct effects, and (c) indirect effects. In what follows, these relations will be specified as a series of hypotheses.

#### Sex-related online behaviors in context (baseline associations)

It is increasingly acknowledged that adolescents’ selection and use of media is an active and context-dependent process [21]. According to the Media Practice Model [22, 23], young people’s media choices are the result of a set of demographic (e.g., gender, age), personal (e.g., interests, experiences), and sociocontextual (e.g., family, peers) orientations. That is, youth select and use media that fit with who they are and what is salient to them at a particular moment. This is also true for their online behavior. Specifically, studies have shown that with more sexual experience, adolescents report using SEIM more frequently [7, 8, 24, 25]. Similarly, adolescents were found to use sexually explicit content more often when they perceived sexual behavior, or exposure to media content involving sexual behavior, to be common or valued among their peers [24, 26, 27]. Based on these findings, we hypothesized the following:

Hypothesis 1a: At baseline, adolescents who have more experience with sexual behavior will use SEIM more frequently.

Hypothesis 1b: At baseline, adolescents who perceive their peers to be more approving of sexual behavior (i.e., injunctive norms) and to be more sexually active (i.e., descriptive norms) will use SEIM more frequently.

Empirical studies on the psychosexual correlates of SNS use are rare. However, in a recent study on adolescent sexual reference display on Facebook and the factors associated with such display, it was found that those displaying sexual references were more engaged in Facebook than their non-displaying peers. In addition, displayers reported more experience with sexual behavior and stronger perceptions that peers are approving of sexual behavior and engaging in sexual activity [5]. These findings are in line with the idea that SNSs may serve as important venues for sexual self-expression among adolescents [4, 6]. Therefore, we hypothesized:

Hypothesis 1c: At baseline, adolescents who have more experience with sexual behavior will spend more time on SNSs.

Hypothesis 1d: At baseline, adolescents who perceive their peers to be more approving of sexual behavior (i.e., injunctive norms) and to be more sexually active (i.e., descriptive norms) will spend more time on SNSs.

#### Sex-related online behaviors predict sexual behavior (direct effect I)

Our integrative model assumes that receptive and interactive sex-related online behaviors directly and uniquely predict adolescents’ subsequent level of experience with sexual behavior. Here, it is important to note that by controlling for baseline levels of experience, the model hypothesizes over-time increases in sexual behavior following engagement in sex-related online behaviors. A theoretical perspective that explains how sex-related online behaviors may predict subsequent sexual behavior is Social Cognitive Theory [28]. Specifically, this theory postulates that people adopt new behaviors by observing the behaviors of significant role models. This observational learning or behavioral modeling is especially likely to occur when (a) behaviors displayed are relevant to the observer, (b) role models are similar to the observer (e.g., same gender or age), (c) role models are attractive or high in status, and (d) role models seem to benefit from displaying the behavior [21, 28]. Hence, through the observation of attractive online models, adolescents may come to learn which behaviors are rewarding. Such behaviors are not necessarily modeled immediately, but instead stored as behavioral scripts that may be retrieved and applied when circumstances evoke it [21, 29]. With regard to SEIM use, social cognitive theory predicts that when sexually interested adolescents repeatedly observe attractive characters enjoying sex with few negative consequences, they will perceive this behavior as rewarding and consequently feel motivated to engage in sexual activities themselves. Hence, we hypothesized:

Hypothesis 2a: More frequent SEIM use will predict increased levels of experience with sexual behavior.

Compared to SEIM, social networking sites are less explicitly sexual in nature; adolescents using SNSs will therefore be less likely to observe and, eventually, internalize visual displays of attractive models engaging in sexual behavior. Instead, behavioral modeling on SNSs may take place through the observation of sexuality as a prominent and valued theme. That is, if notions of sex or discussions of sexual practices on SNSs are common, positively reinforced (e.g., through comments or ‘likes’), and created or shared by age-mates, they may increase adolescents’ positive outcome expectancies regarding sex and promote engagement in sexual behavior [6, 28, 30]. In addition to observational learning and behavioral modeling, social networking sites themselves may increase sexual opportunities. Various studies have suggested that some adolescents use SNSs to broadcast romantic and/or sexual intentions, to initiate romantic relationships, or to find sexual partners [4, 6, 31, 32]. On the basis of these notions, we hypothesized:

Hypothesis 2b: More frequent SNS use will predict increased levels of experience with sexual behavior.

#### Sex-related online behaviors predict perceived peer norms (direct effects II)

Following multisystemic conceptualizations of sexual development [16], we hypothesize that engagement in receptive and interactive sex-related online behaviors influences adolescents’ perceived peer norms regarding sexuality. Scholars have generally argued that, due to its one-sided character, frequent exposure to sexualized media content may shape adolescents’ perceptions of the world around them [21]. This idea is rooted in Cultivation Theory [33], which argues that consistent media portrayals form a specific and biased representation of reality that, after cumulative exposure, may overrule information from other socializing agents such as parents or peers. Over time then, adolescents may gradually “cultivate” or adopt beliefs about the “real world” that are consistent with media’s representation. These beliefs may also include assumptions about the acceptance and prevalence of sexual behavior among peers. Several studies–most of which have employed cross-sectional designs–have indicated that adolescents exposed to sexualized content in traditional media (e.g., television, magazines) offer higher estimates of the numbers of sexually experienced peers [34–36]. This tendency may likely extend to adolescents using SEIM. Specifically, if SEIM portrays sex as common, fun, and risk free, frequent exposure to it may cultivate perceptions that sexual behavior is prevalent and acceptable–that “everyone is doing it” [21]. Therefore, we hypothesize:

Hypothesis 3a: More frequent SEIM use will predict increased perceptions that peers are approving of sexual behavior (i.e., injunctive norms).

Hypothesis 3b: More frequent SEIM use will predict increased estimates of the numbers of peers that have experience with sexual behavior (i.e., descriptive norms).

There is reason to expect that adolescents’ perceived peer norms regarding sexual behavior also change as a result of their SNS use. Research has indicated that aspects of media involvement, such as identification with media models and perceived realism, may influence adolescents’ perceptions over and above amounts of exposure to sexualized content [6, 37]. Given that most content on SNSs is created by adolescents’ peers, identification and perceived realism may be more profound for SNS use. Indeed, prior work has indicated that youth tend to perceive references to substance use and sexuality on SNSs as accurately reflecting real-life attitudes and behaviors [38, 39]. In combination with the large amounts of time adolescents spend on SNSs [5, 30], this led us to hypothesize:

Hypothesis 3c: More frequent SNS use will predict increased perceptions that peers are approving of sexual behavior (i.e., injunctive norms).

Hypothesis 3d: More frequent SNS use will predict increased estimates of the numbers of peers that have experience with sexual behavior (i.e., descriptive norms).

#### Perceived peer norms predict sexual behavior (direct effects III)

As noted earlier, research has consistently demonstrated that adolescents’ sexual decision-making is influenced by their beliefs about prevailing peer norms [19]. This process is described in Social Norms Theory [40], which states that individuals regulate their behavior in concordance with their perceptions of what is common, accepted, or expected among significant referents. These so-called social norms operate as normative pressures and outcome expectancies in guiding behavioral decisions. That is, through perceptions of peers’ approval of sexual behavior (i.e., injunctive norms) adolescents come to learn whether sexual behavior is accepted and/or expected, and through perceptions of peers’ engagement in sexual behavior (i.e., descriptive norms) they evaluate whether sexual behavior is rewarding and therefore beneficial to initiate [40, 41]. It is important to note that injunctive and descriptive norms are based on youths’ subjective beliefs about peers’ approval of and engagement in certain behaviors, and therefore may be misperceptions of actual peer norms. We hypothesized:

Hypothesis 4a: Stronger perceptions that peers are approving of sexual behavior (i.e., injunctive norms) will predict increased levels of experience with sexual behavior.

Hypothesis 4b: Higher estimates of the numbers of peers that are engaging in sexual behavior (i.e., descriptive norms) will predict increased levels of experience with sexual behavior.

Studies investigating the role of perceived peer norms in adolescent sexual (risk) behavior have shown that adolescents’ sexual activity is more strongly related to what they believe their peers do than to what they believe their peers approve of [13, 19]. Although the literature on social norms provides no clear hypothesis or explanation for this difference between descriptive and injunctive norms, it has been suggested that perceptions of peers’ engagement in sexual behavior carry an important additional informational component about the extent to which it is acceptable to engage in sexual behavior [13, 19]. That is, adolescents may assume that peers who engage in sexual behavior also approve of such behavior and of others doing so, whereas they may not be fully aware of the approval of sexual behavior among peers who are not sexually active. On the other hand, it has been argued that if injunctive norms are conceptualized as experienced pressure to engage in a specific behavior (i.e., the extent to which engaging in the behavior is perceived as expected by peers), injunctive norms may be more influential in adolescents’ own behavior [41]. Given these contrasting explanations, we had no hypotheses about the relative importance of injunctive and descriptive norms in predicting adolescents’ level of experience with sexual behavior.

#### Perceived peer norms as mediating processes (indirect effects)

If hypotheses 3a-d and 4a+b are supported, their respective pathways may be combined to form a set of indirect effects; that is, from sex-related online behaviors, through perceived peer norms, to subsequent levels of experience with sexual behavior. Specifically:

Hypothesis 5a: More frequent SEIM use will lead to increased levels of experience with sexual behavior by increasing perceptions of peer approval of sexual behavior (i.e., injunctive norms). [Hypothesis 5c for SNS use]

Hypothesis 5b: More frequent SEIM use will lead to increased levels of experience with sexual behavior by increasing estimates of the numbers of sexually active peers (i.e., descriptive norms). [Hypothesis 5d for SNS use]

Evidence for such indirect effects has been found in studies investigating the link between exposure to sexualized content in traditional media and adolescents’ sexual intentions and behaviors [36, 42]. However, these studies either employed cross-sectional designs or failed to control for baseline levels of perceived peer norms and behavior, rendering them unable to test temporal processes. Moreover, to the authors’ knowledge, no studies have assessed whether perceived peer norms mediate effects of SEIM use and SNS use on subsequent sexual behavior.

### Gender

Some of the key processes in our integrative model may be dependent on adolescents’ gender. It is generally acknowledged that adolescent boys and girls are socialized towards different sexual scripts. This gender-specific sexual socialization is deeply affected by a phenomenon described as the "sexual double standard," which refers to the acceptance of a set of norms prescribing sexual attractiveness yet sexual modesty for girls, while praising sexual assertiveness and permissiveness for boys [43–45]. The sexual double standard may lead to conflicting beliefs about prevailing norms regarding sexuality, where sexual activity is expected for boys but disapproved of for girls [46]. Different socialization messages may also influence the types of online behaviors boys and girls engage in, and the way they process and respond to media content [22, 23, 47]. For instance, it has been proposed that boys are more likely to use SEIM and more likely to be influenced by its content because SEIM portrays sex in a way that for boys may be socially acceptable, whereas it generally contrasts with prevailing socialization scripts for girls [48]. Given these potential gender differences, we tested our integrative model for boys and girls separately.

## Method

### Participants

Data for this study were collected as part of Project STARS, a longitudinal research project on romantic and sexual development of Dutch adolescents. A convenience sample of adolescents in grades six through ten were followed up across four waves, with six-month intervals between waves. The first measurement wave (T_1_) was conducted in the Fall of 2011. The longitudinal sample consisted of 1,297 participants (53.3% boys). For the present study, only seventh to tenth grade students (*n* = 1,132) were included as the questionnaire for the sixth grade students did not contain all investigated concepts. At T_1_, this sample (52.7% boys) had an average age of 13.95 years (*SD* = 1.18; range 11–17). Most participants (79.2%) had a Dutch background (self and both parents born in the Netherlands); 11.0% had another Western background (self or a parent born in Europe, US, Canada, Australia, or New-Zealand), and 9.8% had a non-Western background (self or a parent born in an African, Middle Eastern, Asian, or South-American country). Adolescents were enrolled in different educational tracks, with approximately 40% in vocational education programs and 60% in college or university preparatory programs.

Because of school absence on the day of measurement and the graduation of several tenth graders after T_2_, some of our participants were not able to complete all four questionnaires. Of 1,132 participants, 815 (72.0%) contributed data at all four waves. At T_1_, T_2_, T_3_, and T_4,_ the number of participants was 1,066 (94.2%), 1,047 (92.5%), 1,010 (89.2%), and 925 (81.7%), respectively. Compared to participants who completed all questionnaires, participants who missed one or more measurement waves were more often boys, *χ*²(1, *N* = 1,132) = 10.21, *p* = .001, older, *t*(503.21) = -6.71, *p* < .001, enrolled in lower educational levels, *χ*²(1, *N* = 1,065) = 66.80, *p* < .001, and more often had a non-Western background, *χ*²(1, *N* = 1,132) = 12.55, *p* < .001. Moreover, they reported higher levels of SEIM use, *t*(314.96) = -5.00, *p* < .001, injunctive and descriptive peer norms, *t*_injunctive_(363.54) = -8.55, *p* < .001 respectively *t*_descriptive_(342.64) = -8.26, *p* < .001, and sexual experience, *t*(295.59) = -8.04, *p* < .001, at the start of the study. It should be noted that our data-analysis procedure (full information maximum likelihood, a common procedure to handle missing data) includes cases with partially missing data; therefore, our results are based on the complete sample [49].

### Procedure

Adolescents were recruited from schools in large cities and small municipalities throughout the Netherlands. Schools were randomly approached, yet purposefully selected from different areas of the Netherlands. Interested schools were visited by the researchers for a personal meeting with the principal, during which the study goals and procedures were introduced and explained. Eventually, four secondary schools agreed to participate. The school principals and researchers decided together which classes within the school would be selected for participation.

Prior to the first measurement, both adolescents and their parents received letters, brochures, and flyers describing the aims of the study and the possibility to decline or end participation at any time. Parents could return signed forms indicating that their child was not allowed to take part in the study (6.9% of the approached parents did so). Adolescents with passive informed parental consent were ensured at each measurement occasion that participation was voluntary and that they could return to their classroom if they did not wish to take part in the study (0.1% did so).

At each wave, adolescents completed a computer-based, Dutch questionnaire at school during regular school hours. Researchers and trained research assistants were present to supervise the data collection (i.e., introduce the project and the procedure, answer questions, and ensure maximum privacy from teachers and other students). Teachers were not present in the classroom during the data collection. Confidentiality of responses was guaranteed, as was the option to stop participation at any time. Adolescents received book gift certificates of increasing values after each completed questionnaire. An ethical protocol was developed should participants have any problems or questions concerning issues in this study. The ethics board of the Faculty of Social and Behavioural Sciences of Utrecht University approved all study and consent procedures.

### Measures

#### Experience with sexual behavior (T_1_ and T_4_)

To assess adolescents’ experience with sexual behavior, participants initially were asked two questions: “Have you ever French kissed somebody?” and “Have you ever had sex with another person? With sex we mean everything from touching or caressing to intercourse,” (0 = No, 1 = Yes). Those who indicated Yes on the second question received follow-up questions about their experience with different sexual behaviors: naked touching or caressing, performing or receiving manual sex, performing or receiving oral sex, and vaginal or anal intercourse (0 = No, 1 = Yes). The kissing and sexual behavior items were combined into one variable measuring the level of adolescents’ experience with sexual behavior, ranging from 0 = Inexperienced with all five behaviors to 5 = Experience with five behaviors (Cronbach’s α_T1_ = .78; α_T4 =_ .86).

#### Sex-related online behaviors (T_1_)

SEIM use. Based on research on the wording of sensitive questions [50], adolescents’ SEIM use was assessed as follows: “Many teenagers sometimes look at pornography on the Internet. We would like to know how this is for you. How often do you use the Internet to view a porn website (a website with pictures or movies that show nudity or people having sex)?” The response categories for this item were 1 = Never, 2 = Less than once a year, 3 = Less than once a month, 4 = One to three times a month, 5 = Once or twice a week, 6 = Three times a week or more.

SNS use. Adolescents’ use of SNSs was measured by asking participants how much time they actively spent each day on their most used social networking site. Response categories were 0 = Not an SNS member, 1 = Less than 15 minutes, 2 = 15–30 minutes, 3 = 30–60 minutes, 4 = 1–2 hours, 5 = 3–4 hours, and 6 = More than 4 hours.

#### Perceived peer norms (T_1_ and T_3_)

Injunctive norms. Adolescents’ perceptions of their peers’ approval of sexual behavior were measured with an adapted version of an item previously used to assess parental approval of sexual behavior [51]. This item read: “My best friends believe that boys and girls our age should not have sex yet”, scored on a six-point scale (1 = Completely not true, 6 = Completely true). Scores were reversed, so that a higher score indicated that adolescents perceived their peers to be more approving of sexual behavior.

Descriptive norms. Adolescents’ perceptions of their peers’ experience with sexual behavior were measured with three items pertaining to the proportion of friends adolescents thought had experience with French kissing, sexual intercourse, and one-night stands [52,53], scored on a six-point scale (1 = None of my friends, 2 = Only a few of my friends, 3 = Less than half of my friends, 4 = More than half of my friends, 5 = Almost all of my friends, 6 = All of my friends). A composite score was created by averaging the scores on these items (α_T1_ = .72; α_T3_ = .73).

#### Strategy of analysis

The conceptual model presented in Fig 1 was tested using structural equation modeling in Mplus (Version 7.2; [54]). We estimated two models, one including SEIM use and one including SNS use. Sex-related online behaviors were measured at baseline (T_1_); perceived peer norms and experience with sexual behavior were measured both at baseline and at 12 (T_3_) and 18 (T_4_) months follow-up, respectively. This way, actual over-time change in peer norms and sexual behavior following engagement in sex-related online behaviors could be assessed. Age was included in the models as a control variable and models were estimated for boys and girls separately.

We used a bootstrap procedure to estimate models as this alleviates problems with significance testing when normality assumptions are violated [55]–a typical phenomenon in sex research. We obtained 1,000 bootstrap samples and analyzed 95% bias-corrected confidence intervals for all hypothesized effects. If these intervals do not include the value zero, the estimated effect is significant. We considered an effect as significant only if both its *p*-value and its 95% bias-corrected confidence interval indicated a statistically significant difference from zero. Model fits were evaluated with the Comparative Fit Index (CFI) and the Root Mean Square Error of Approximation (RMSEA). CFIs greater than .90 and RMSEAs less than .08 were considered as evidence of an adequate model fit [56].

To analyze whether adolescents’ SEIM use and SNS use predicted, through increased perceptions of peer approval and activity, increased levels of experience with sexual behavior (H5), we evaluated the significance of indirect effects generated with the product-of-coefficients method [54, 57].

## Results

### Descriptives and preliminary analyses

Descriptive statistics for the key variables are shown in Table 1. Sex-related online behaviors varied significantly for boys and girls: boys reported more frequent SEIM use than girls, whereas girls spent more time per day on SNSs. With regard to perceived peer norms, boys were found to report stronger perceptions that peers were approving of and engaging in sexual behavior than girls, both at baseline (T_1_) and at 12 months follow-up (T_3_). Pairwise *t* tests further demonstrated that for both boys’ and girls’ these peer norms significantly increased over the 12 month interval (boys: *t*_injunctive_(474) = -10.63, *p* < .001, *t*_descriptive_(413) = -4.96, *p* < .001; girls: *t*_injunctive_(453) = -8.80, *p* < .001, *t*_descriptive_(417) = -6.99, *p* < .001). Baseline levels of experience with sexual behavior were somewhat higher for boys compared to girls; however, this difference was no longer apparent at T_4_. As expected, boys’ and girls’ level of experience with sexual behavior increased during the 18 month period between T_1_ and T_4_ (boys: *t*(434) = -9.69, *p* < .001; girls: *t*(437) = -10.44, *p* < .001). Table 2 shows the correlation coefficients of the variables included in the integrative model. As this table shows, sex-related online behaviors, perceived peer norms, and experience with sexual behavior were all positively correlated (with the exception of girls’ SEIM use and T_3_ injunctive norms).

**Table 1:** Descriptive Statistics for Key Variables in the Integrative Model for Boys and Girls.

		Boys	Girls	
	range	*M* (*SD*)	*M* (*SD*)	Gender difference
1. SEIM use (frequency) (T_1_)	1–6	2.42 (1.71)	1.10 (0.44)	*t* = 17.60***
2. SNS use (time per day) (T_1_)	0–6	2.39 (1.53)	2.91 (1.42)	*t* = -5.72***
3. Injunctive norms (T_1_)	1–6	3.37 (1.84)	2.72 (1.69)	*t* = 6.01***
4. Injunctive norms (T_3_)	1–6	4.15 (1.75)	3.35 (1.80)	*t* = 7.11***
5. Descriptive norms (T_1_)	1–6	2.25 (1.22)	2.03 (1.05)	*t* = 2.99**
6. Descriptive norms (T_3_)	1–6	2.44 (1.28)	2.25 (1.12)	*t* = 2.43*
7. Sexual behavior (T_1_)	0–5	0.74 (1.24)	0.57 (1.00)	*t* = 2.46*
8. Sexual behavior (T_4_)	0–5	1.06 (1.52)	1.08 (1.52)	*t* = 1.47

SEIM = Sexually Explicit Internet Material; SNS = Social Networking Site.

* *p* < .05

** *p* < .01

*** *p* < .001 (two-tailed).

**Table 2:** Pearson Correlations between Key Variables in the Integrative Model for Boys and Girls.

	1.	2.	3.	4.	5.	6.	7.	8.
1. SEIM use (T_1_)	-	.01	.14**	.08	.15**	.10*	.23***	.20***
2. SNS use (T_1_)	.22***	-	.25***	.29***	.37***	.30***	.24***	.28***
3. Injunctive norms (T_1_)	.46***	.22***	-	.54***	.53***	.45***	.42***	.38***
4. Injunctive norms (T_3_)	.36***	.25***	.51***	-	.55***	.60***	.40***	.48***
5. Descriptive norms (T_1_)	.44***	.29***	.50***	42***	-	.66***	.58***	.53***
6. Descriptive norms (T_3_)	.35***	.24***	.41***	.47***	.58***	-	.47***	.48***
7. Sexual behavior (T_1_)	.43***	.25***	.45***	.37***	.59***	.48***	-	.60***
8. Sexual behavior (T_4_)	.38***	.32***	.38***	.40***	.49***	.54***	.65***	-

Correlation coefficients for boys are presented below the diagonal; correlation coefficients for girls are presented above the diagonal.

SEIM = Sexually Explicit Internet Material; SNS = Social Networking Site.

* p < .05

** p < .01

*** p < .001 (two-tailed).

### Analysis of the integrative model

**Fig 2:**
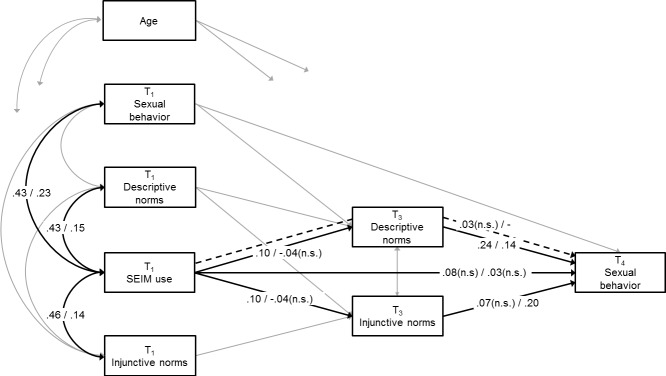
Estimated model for SEIM use. Values are standardized regression coefficients, significant at least at *p* < .05 (two-tailed), unless indicated otherwise. Values before the slash represent estimates for boys; values behind the slash represent estimates for girls. Model fit boys: Comparative Fit Index = 1.00, Root Mean Square Error of Approximation = .04. Model fit girls: Comparative Fit Index = .99, Root Mean Square Error of Approximation = .08.

**Fig 3:**
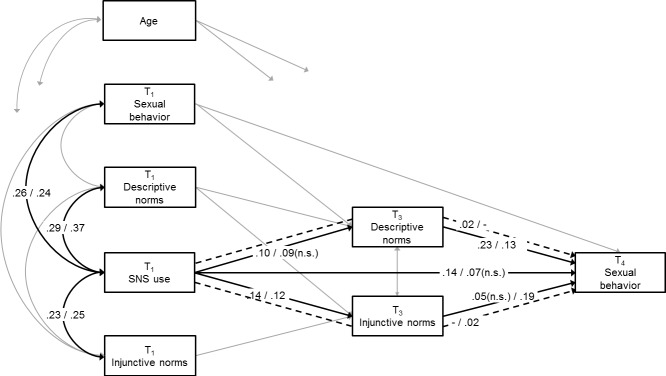
Estimated model for SNS use. Values are standardized regression coefficients, significant at least at *p* < .05 (two-tailed), unless indicated otherwise. Values before the slash represent estimates for boys; values behind the slash represent estimates for girls. Dashed line represents indirect effect. Model fit boys: Comparative Fit Index = 1.00, Root Mean Square Error of Approximation = .04. Model fit girls: Comparative Fit Index = .99, Root Mean Square Error of Approximation = .06.

Our initial models did not show adequate fit (i.e., all RMSEAs > .10). Inspection of the modification indices revealed that two additional pathways had to be included in the models in order to fit the data. Specifically, adding paths from (1) T_1_ sexual behavior to T_3_ descriptive norms and (2) T_1_ descriptive norms to T_3_ injunctive norms resulted in models with acceptable fit, CFIs ≥ .99; RMSEAs ≤ .08. The final models for SEIM use and SNS use are presented in Figs 2 and 3, respectively. To emphasize the results of most interest, these figures only present coefficients for the hypothesized and theoretically described relations. Direct effects from the covariates (age and baseline levels of peer norms and sexual behavior) to the key variables are excluded from the figure, as are the remaining concurrent associations. These paths were positive and mostly significant, with the exception of: (a) age with SEIM use (girls), (b) age with SNS use (boys and girls), (c) age to T_3_ descriptive norms (boys), (d) age to T_4_ sexual behavior (boys and girls); non-significant effects ranged from *B* = 0.03 (*β* = .02) to *B* = 0.09 (*β* = .08). The integrative models accounted for 59% and 61% of the variance in boys’ level of experience with sexual behavior and 50% and 51% of the variance in girls’ level of experience with sexual behavior.

#### Baseline associations

As predicted in Hypothesis 1a, adolescents who had more baseline experience with sexual behavior reported more frequent SEIM use (boys: *B* = 0.92, *β* = .43, *p* < .001, bc 95% CI [0.71, 1.15]; girls: *B* = 0.10, *β* = .23, *p* = .008, bc 95% CI [0.03, 0.18]). Moreover, in line with Hypothesis 1b, adolescents who reported more perceived peer approval of and peer engagement in sex at the start of the study used SEIM more frequently (boys: *B*_injunctive_ = 1.43, *β* = .46, *p* < .001, bc 95% CI [1.18, 1.69], *B*_descriptive_ = 0.89, *β* = .43, *p* < .001, bc 95% CI [0.70, 1.08]; girls: *B*_injunctive_ = 0.10, *β* = .14, *p* = .002, bc 95% CI [0.05, 0.18], *B*_descriptive_ = 0.07, *β* = .15, *p* = .002, bc 95% CI [0.03, 0.11]). The same patterns were found for SNS use, confirming Hypothesis 1c (boys: *B* = 0.49, *β* = .26, *p* < .001, bc 95% CI [0.30, 0.68]; girls: *B* = 0.34, *β* = .24, *p* < .001, bc 95% CI [0.21, 0.50]) and Hypothesis 1d (boys: *B*_injunctive_ = 0.63, *β* = .23, *p* < .001, bc 95% CI [0.38, 0.87], *B*_descriptive_ = 0.54, *β* = .29, *p* < .001, bc 95% CI [0.37, 0.69]; girls: *B*_injunctive_ = 0.59, *β* = .25, *p* < .001, bc 95% CI [0.35, 0.81], *B*_descriptive_ = 0.54, *β* = .37, *p* < .001, bc 95% CI [0.41, 0.70]).

#### Direct effects

Hypothesis 2a stated that more frequent SEIM use would directly predict increased levels of experience with sexual behavior. This hypothesis had to be rejected (boys: *B* = 0.08, *β* = .08, *p* = .120, bc 95% CI [-0.03, 0.17]; girls: *B* = 0.10, *β* = .03, *p* = .647, bc 95% CI [-0.36, 0.46]). Hypothesis 2b, predicting that more frequent SNS use would lead to increased levels of experience with sexual behavior, received support for boys (boys: *B* = 0.16, *β* = .14, *p* < .001, bc 95% CI [0.08, 0.23]; girls: *B* = 0.08, *β* = .07, *p* = .099, bc 95% CI [-0.02, 0.17]). More frequent SNS use predicted increases in boys’ level of experience with sexual behavior 18 months later.

Hypotheses 3a and 3b predicted that more frequent SEIM use would increase adolescents’ perceptions that peers are approving of and engaging in sexual behavior. These over-time effects were indeed found, albeit for boys only (boys: *B*_injunctive_ = 0.10, *β* = .10, *p* = .020, bc 95% CI [0.10, 0.18], *B*_descriptive_ = 0.08, *β* = .10, *p* = .028, bc 95% CI [0.01, 0.15]; girls: *B*_injunctive_ = -0.15, *β* = -.04, *p* = .425, bc 95% CI [-0.56, 0.20], *B*_descriptive_ = -0.09, *β* = -.04, *p* = .479, bc 95% CI [-0.32, 0.21]). Hypotheses 3c and 3d, which predicted that more frequent SNS use would increase adolescents’ perceptions that peers are approving of and engaging in sexual behavior, were partially supported. Specifically, boys’ SNS use predicted increases in their injunctive and descriptive norms 12 months later, whereas girls’ SNS use predicted increases in their injunctive norms, but only marginally in their descriptive norms (boys: *B*_injunctive_ = 0.17, *β* = .14, *p* < .001, bc 95% CI [0.08, 0.25], *B*_descriptive_ = 0.08, *β* = .10, *p* = .010, bc 95% CI [0.02, 0.15]; girls: *B*_injunctive_ = 0.15, *β* = .12, *p* = .003, bc 95% CI [0.05, 0.25], *B*_descriptive_ = 0.07, *β* = .09, *p* = .051, bc 95% CI [0.00, 0.15]).

As expected in Hypotheses 4a and 4b, perceived peer norms regarding sexuality positively predicted adolescents’ experience with sexual behavior. For boys, stronger perceptions that peers are engaging in sex predicted increased levels of experience with sexual behavior six months later (*B*_descriptive_ = 0.29, *β* = .23, *p* < .001, bc 95% CI [0.17, 0.45]); however, the effect of injunctive norms on subsequent sexual behavior did not reach significance (*B*_injunctive_ = 0.05, *β* = .05, *p* = .211, bc 95% CI [-0.02, 0.13]). For girls, stronger perceptions that peers are approving of and engaging in sex predicted increased experience with sexual behavior six months later (*B*_injunctive_ = 0.16, *β* = .19, *p* < .001, bc 95% CI [0.09, 0.25], *B*_descriptive_ = 0.18, *β* = .13, *p* = .022, bc 95% CI [0.03, 0.35]). (These estimates are derived from the SNS models; estimates from the SEIM model may slightly differ but do not alter the conclusions.)

#### Indirect effects

On the basis of the above findings, we assessed three different pathways through which sex-related online behaviors may indirectly increase adolescents’ experience with sexual behavior. For the first pathway, which represented the effect of boys’ SEIM use on subsequent sexual behavior through descriptive norms, the indirect effect did not reach significance (*B* = 0.02, *β* = .03, *p* = .066, bc 95% CI [0.00, 0.06]). However, for the second pathway, representing the effect of boys’ SNS use on sexual behavior through descriptive norms, the indirect effect appeared significant (*B* = 0.03, *β* = .02, *p* = .031, bc 95% CI [0.01, 0.05]). Similarly, results for the third pathway, constituting the effect of girls’ SNS use on sexual behavior through injunctive norms, showed a significant indirect effect (*B* = 0.03, *β* = .02, *p* = .018, bc 95% CI [0.01, 0.05]). Hence, in line with Hypotheses 5c and 5d, SNS use predicted increased levels of experience with sexual behavior by increasing perceptions that peers are engaging in sexual behavior among boys and perceptions that peers are approving of sexual behavior among girls.

## Discussion

The current study aimed to take an integrative approach to better understand how sex-related online behaviors and peer influences interrelate and combine in shaping adolescents’ sexual development. Specifically, we tested an integrative model explaining how receptive (i.e., SEIM use) and interactive (i.e., SNS use) sex-related online behaviors are linked to perceived peer norms in predicting adolescents’ level of experience with sexual behavior.

Our findings contributed to the literature about the role of sex-related online behaviors in adolescents’ sexual development in several ways. Firstly, our results showed that sex-related online behaviors are indeed interrelated with sex-related processes in the peer domain. Specifically, adolescents who used SEIM more often and who spent more time on SNSs were also more likely to perceive their peers to be approving of sexual behavior (i.e., injunctive norms) and to be sexually active (i.e., descriptive norms). Moreover, both adolescents’ sex-related online behaviors and their perceived peer norms were concurrently associated with higher levels of experience with sexual behavior.

A second contribution of our findings is that they illustrate the different pathways through which sex-related online behaviors predict adolescents’ experience with sexual behavior. Our model showed that among boys, more time spent on SNSs directly predicted increased levels of experience with sexual behavior 18 months later. This direct effect was not found for girls, despite the finding that on average girls reported more frequent SNS use. Moreover, no direct effects of adolescents’ SEIM use on their subsequent experience with sexual behavior were identified. However, sex-related online behaviors particularly predicted increases in adolescents’ level of experience with sexual behavior by affecting their perceptions of peer norms toward sexuality. Specifically, boys who used SEIM more often and who spent more time on SNSs showed increases over time in their beliefs that peers are approving of sexual behavior and in their estimates of the numbers of sexually active peers. Similarly, girls who spent more time on SNSs reported increases in their perceptions of peers’ approval of sexual behavior (and marginally in their estimates of the numbers of sexually active peers). These perceptions (i.e., descriptive norms for boys, injunctive and descriptive norms for girls), in turn, predicted increased levels of experience with sexual behavior. Although the point estimates of the indirect effects were small (and non-significant in the case of boys’ SEIM use and girls’ SNS use through descriptive norms), these findings show that both receptive and interactive sex-related online behaviors have the potential to alter adolescents’ perceptions of what is common and accepted, probably resulting in increased normative pressure and/or more positive outcome expectancies for engaging in sexual behavior [40]. As such, our study confirms theoretical notions of Cultivation Theory and Social Norms Theory that sexual decision-making is particularly influenced by perceived normative behavior, and that media content may shape those critical perceptions [19, 33, 40]. Furthermore, our findings build on previous research demonstrating that exposure to sexualized media content predicts adolescents’ sexual behavior by changing their perceptions of peer sexual norms [36, 42]. Importantly, our findings suggest that this may be particularly true for SNS use–an increasingly popular behavior that is more social than explicitly sexual–and therefore confirm the need to jointly consider the multiple influencing systems in adolescents’ sexual development.

A third contribution of our findings is that they highlight important gender differences in how sex-related online behaviors may predict subsequent sexual behavior. Firstly, in contrast to boys, girls’ SEIM use was not related to changes over time in their perceptions of peer norms toward sexuality. This finding could reflect girls’ lower exposure to SEIM, which may be insufficient to cultivate perceptions about the acceptance and prevalence of sexual behavior [21, 33]. It could be that girls who use SEIM experience a sense of “false uniqueness”, that is, they believe that their use of SEIM is idiosyncratic and non-normative among their female peers [58]. Because they view themselves as deviant, they may be less likely to associate SEIM’s representations of sexuality with their own and peers’ reality. On a related note, the lack of effects for girls may be explained in terms of the nature of SEIM. That is, SEIM portrays sexual encounters predominantly in a male-oriented manner that may correspond with prevailing sexual scripts for boys (i.e., sexual assertiveness), yet may contrast with prevailing scripts for girls (i.e., sexual modesty, girls as gatekeepers; [43–45]). Girls, then, may need to use SEIM more frequently in order to overrule these prevailing scripts and change their existing beliefs. Secondly, our findings show that different perceived peer norms may be dominant in the effects of boys’ and girls’ SNS use on their subsequent experience with sexual behavior. Although boys’ SNS use shaped both types of perceived peer norms, it was the increases in their estimates of the numbers of sexually active peers that subsequently predicted increases in their own levels of experience with sexual behavior. In contrast, girls’ SNS use predicted increased levels of experience with sexual behavior particularly by increasing their beliefs about peers’ approval of sex. This difference seems to reflect the gendered sexual socialization scripts in which (dis)approval of sexuality is a major theme for girls, whereas sexual assertiveness is emphasized for boys [46]. It also raises important questions about the specific content boys and girls are exposed to on SNSs. For instance, it could be that girls encounter more sex-positive attitudes on SNSs, which allow them to feel more comfortable exploring their sexuality. At the same time, the marginally significant effect of girls’ SNS use on their subsequent descriptive norms requires further examination, especially given its predictive role in girls’ sexual behavior. Together, these findings point to the subtleties that characterize media influence and the importance of examining the (gender-)specific messages adolescents create, post, and are exposed to when they engage in receptive and interactive sex-related online behaviors [2].

Despite these valuable contributions, some limitations of our study design should be noted. First, although our longitudinal model enabled us to test hypotheses drawn from social cognitive theory, cultivation theory, and social norms theory about the temporal sequence in which adolescents’ sex-related online behaviors, perceived peer norms, and sexual behavior are related, other pathways of influence may exist. For instance, the time lag between the measurement of sex-related online behaviors and adolescents’ level of experience with sexual behavior in our study may have been too large to identify more direct effects between these constructs. Second, we have no information about the specific content adolescents were exposed to when they engaged in sex-related online behaviors. To understand more accurately why sex-related online behaviors are associated with changes in perceived peer norms and, eventually, with increases in sexual behavior, it is necessary to examine the nature of the messages adolescents encounter online. Although we do have consistent content-analytic evidence about prevailing portrayals of sexuality in SEIM [59], such knowledge is insufficiently available when it comes to messages on SNSs. It is important in this regard to also take into account the different purposes of different SNSs. Recently developed location-based SNSs such as Grindr and Tinder are more specifically targeted toward finding romantic and sexual partners, and may therefore differentially relate to perceived peer norms and sexual behavior. Third, our study focused on SEIM use and SNS use as indicators of adolescents’ sex-related online behaviors. Future studies should expand our findings by testing integrative models with other online behaviors, such as sexual information-seeking and cybersex. Future studies should also examine how sex-related online behaviors interrelate and interact with other domains of influence, such as the self and the family system, in predicting adolescent sexual development. On a related note, scholars from both media and peer relations traditions have argued that media and peer effects are conditional–that some adolescents are more susceptible to their influences than others [60, 61]. To inform and guide prevention and intervention efforts, research should aim to identify moderating factors that amplify or attenuate effects of media content or peer norms on adolescents’ sexuality. Fourth, we measured perceived peer norms regarding sexuality among adolescents’ (best) friends. Future studies should examine whether adolescent sexual development is differentially related to perceived norms among different types of peers, including age-mates in general, high-status peers, more distant online peers, crowds, and romantic or sexual partners [60]. Fifth, we measured the concepts in our integrative model using adolescent self-reports. Although this is still the most common method to collect data on sexuality, it is well-documented that adolescents may underreport their sexual experiences or sex-related media use, due to fear of embarrassment, disapproval, or social sanctions [62]. Finally, our results are based on a convenience sample in the Netherlands. The extent to which our results can be generalized to other populations of adolescents requires further investigation.

## Conclusion

Adolescents’ sexual development is a complex process influenced by multiple interrelating systems. Among these multiple systems of influence, the Internet and peers occupy a particularly prominent role in youths’ daily lives; yet research on adolescents’ sexual development has rarely studied these systems together. The current study tested an integrative model explaining how receptive (i.e., SEIM use) and interactive (i.e., SNS use) sex-related online behaviors are linked to perceived peer norms in predicting adolescents’ level of experience with sexual behavior. Our findings demonstrate that both types of sex-related online behaviors have the potential to alter adolescents’ perceptions of what is common and accepted, probably resulting in increased normative pressure and/or more positive outcome expectancies for engaging in sexual behavior. As such, they highlight the need for a multisystemic approach to research on adolescents’ sexual development. Moreover, our findings may guide prevention and intervention efforts that aim to promote youths’ sexual health. Such efforts should not only focus on educating youth how to interpret and put into perspective online content, but also on developing skills aimed at reducing susceptibility to perceived norms.
